# Environmental Exposure of Wild Carnivores to Zoonotic Pathogens: *Leptospira* Infection in the First Free Living Wolf (*Canis lupus* Linnaeus, 1758) Found Dead in the Friuli Venezia Giulia Region

**DOI:** 10.3390/ijerph18052512

**Published:** 2021-03-03

**Authors:** Marco Bregoli, Stefano Pesaro, Martina Ustulin, Denis Vio, Paola Beraldo, Marco Galeotti, Monia Cocchi, Laura Lucchese, Cristina Bertasio, Maria Beatrice Boniotti, Luca Lapini, Alda Natale

**Affiliations:** 1 Istituto Zooprofilattico Sperimentale delle Venezie, Viale dell’Università, 35020 Legnaro, Italy; mustulin@izsvenezie.it (M.U.); dvio@izsvenezie.it (D.V.); mcocchi@izsvenezie.it (M.C.); llucchese@izsvenezie.it (L.L.); anatale@izsvenezie.it (A.N.); 2 Dipartimento di Scienze AgroAlimentari, Ambientali e Animali Sezione di Patologia Veterinaria, Università degli Studi di Udine, Via Sondrio, 33100 Udine, Italy; stefano.pesaro@uniud.it (S.P.); paola.beraldo@uniud.it (P.B.); marco.galeotti@uniud.it (M.G.); 3 National Reference Centre for Animal Leptospirosis, Istituto Zooprofilattico Sperimentale della Lombardia ed Emilia Romagna “Bruno Ubertini”, 25121 Brescia, Italy; cristina.bertasio@izsler.it (C.B.); mariabeatrice.boniotti@izsler.it (M.B.B.); 4 Zoology Section of the Friulian Natural History Museum, Via C. Gradenigo Sabbadini, 33100 Udine, Italy; luca.lapini@comune.udine.it

**Keywords:** *Leptospira*, *Canis lupus*, zoonosis

## Abstract

Leptospirosis is a worldwide-spread zoonosis causing disease and death in dogs and in humans. A Leptospiral infection has been recorded in several wild carnivore species in Europe, but tissue pathological changes were not commonly described. The Grey wolf (*Canis lupus*) has been expanding its distribution range in north-eastern Italy during the last decade. A young wolf, representing the first individual handled in the region, was found road-killed and then submitted to necropsy. Pathological changes included erosive lesions of gingival mucosa, mild liver enlargement, and multifocal degenerative-necrotic areas along with hyperemic reactive lesions; multifocal interstitial nephritis and multifocal lung hemorrhages were observed. A Polymerase Chain Reaction (PCR) able to detect pathogenic species of *Leptospira* performed on a kidney sample was positive. Serological reactions for serogroup Gryppotyphosa (1:6400), Pomona (1:800), and Icterohaemorrhagiae (1:200) were evidenced by MAT. Genotyping by Multilocus Sequence Typing (MLST) performed on detected *Leptospira* characterized it as belonging to Sequence Type (ST) 117, which refers to *L. kirschneri*, serogroup Pomona, serovar Mozdok. Regardless of the role of *Leptospira* infection as an eventual predisposing factor to the road killing of this wolf, to the best of the authors’ knowledge, this is the first report of *Leptospira*-induced pathology in a wolf in Europe. Surveys on *Leptospira* infection in free-ranging wildlife species should be pursued in order to achieve further epidemiological knowledge on the circulation of the *Leptospira* strain.

## 1. Introduction

Spirochetes of the genus *Leptospira* are regarded as some of the most important widespread and neglected zoonotic bacterial agents. Many *Leptospira* species are pathogens able to survive for months in the environment, causing infection in humans and animals, and also in some *reservoir* hosts that can shed the bacteria in their urine for a long time. The epidemiology of the disease can vary according to the ecology of cohabiting and interacting species and relating to serovars/genospecies distribution. Evidence of leptospiral infection has been reported in European wild carnivore species such as red fox (*Vulpes vulpes*), pine marten (*Martes martes*), stone marten (*Martes foina*), badger (*Meles meles*), lynx (*Lynx lynx*), brown bear (*Ursus arctos*), and wolf (*Canis lupus*) [1]; all of these species are present in our region. *Leptospira*-induced gross pathology has not been described in European wild carnivores [1]. Serovar Icterohaemorrhagiae and Canicola were recorded in red foxes and golden jackals (*Canis aureus*) respectively [2,3].

The Friuli Venezia Giulia region and northern Italy is an endemic area for leptospirosis. Despite widespread vaccination of dogs (*Canis lupus familiaris*), *Leptospira* serovars are regularly responsible for the disease and death of dogs each year [4,5]. The current regional wildlife surveillance plan includes leptospirosis within priority diseases of wild carnivores and its monitoring is being carried out by means of passive surveillance. The panel of selected pathogens includes also rabies, canine distemper virus, *Echinococcus multilocularis*, *Trichinella* sp., and sarcoptic mange. The wolf has been expanding its distribution range in north-eastern Italy during the last decade; north-eastern Italy includes the lands where Italian wolf (*Canis lupus italicus*) and Dinaric-Balkan wolf populations of the Eurasian wolf (*Canis lupus lupus*) have gathered and mixed after almost 80 years from the extinction in this area [6,7,8], which dated back to 1929 (Campobon, Belluno Province: [6]) or to 1924 (Basovizza, Province of Trieste: [5]). These historic presences were referred to as Balkan or Center-European Eurasian wolves (*Canis lupus lupus* Linnaeus 1758).

## 2. Materials and Methods

A young wolf (7 months old estimated) was found road-killed in October 2018 and then submitted to necropsy, representing the first found dead individual studied since the local extinction in the region. The first age estimation of this young specimen was inferred from a count of the time passed between its birth (April, determined by camera-trapping) and its death. This first age estimation was then confirmed by various osteologic features, examined after its skeletal preparation: the roots of the canines were still completely open, the skeletal joint capsules never fused, the axial cranial sutures never closed. The young wolf studied in the present paper surely was a cross-breeding between an Italian male from Peninsular Italy and a female from Lessinia (Verona) born from a couple composed of a Slovenian male and an Italian female; bio-molecular determination of the familiar relationships of the road-killed young wolf derived from the ongoing project Life WolfAlps [9]. The origin of the road-killed young wolf could be important to understand its parasite-history and pathology.

The subject was a male, 21 kg heavy. Apart from traumatic injuries, pathologic changes were recorded; according to gross pathological lesions and standardized protocols, analyses were conducted including bacteriological (liver and kidney), specific search for *Salmonella* spp. (liver), virological (lungs, liver, and gut by Polymerase Chain Reaction PCR and electron microscopy), and histopathological (liver and kidney), and a blood sample was collected for serological investigations.

Furthermore, fecal and muscle samples for a research project on *E. multilocularis* and Trichinella were stored (RC 18/16 IZSVE—Health Ministry Funds).

Specific tests for leptospirosis were the Micro Agglutination Test (MAT) for the detection of antibodies on serum samples, real-time PCR for antigen search from kidney, and Multilocus Sequence Typing (MLST) for characterization.

The MAT was performed following the Office International des Epizooties (OIE) method (Chap 3.1.12), with a cut-off of 1:100. The antigen panel included 8 serogroups and 11 serovars (sv) distributed by the Italian Reference Center for Animal Leptospirosis as antigens in the routine diagnostic microagglutination test: Icterohaemorrhagiae sv Icterohaemorrhagiae (strain Bianchi), Icterohaemorrhagiae sv Copenagheni (strain Wijnberg n. 1), Australis sv Bratislava (strain Riccio 2 n. 47), Canicola sv Canicola (strain Alarik n. 2), Sejroe sv Hardjo (strain Hardjoprajitno n. 224), Sejroe sv Sejroe (strain M84), Sejroe sv Saxkoebing (strain Mus24), Tarassovi sv Tarassovi (strain Mitis Johnson n. 6), Grippothyphosa sv Grippotyphosa(strain Moska V n. 54), Pomona sv Pomona (strain Pomona n. 222), Ballum sv Ballum (strain Mus 127 n. 217).

With regard to the real-time PCR, a commercially available High Pure PCR Template Preparation kit (Roche Diagnostics, Mannheim, Germany) was used, in accordance with the manufacturer’s instructions, to extract 1 cm^3^ tissue homogenate. The deoxyribonucleic acid (DNA) extraction preparations included a negative control (water). The DNA extracted from the kidney was subjected to a TaqMan-based real-time PCR assay targeting an 87-bp fragment that corresponded to a portion of the gene encoding the 16S rDNA [10]. The PCR was performed in a 25 µL final volume, containing 3 µL of extracted DNA, 12.5 µL of 2× Master Mix TaqMan Universal 2× (Thermo Fisher Scientific, Waltham, MA, USA), 300 nM of each primer, and 100 nM of a 5′ 6-carboxyfluorescein (FAM)–3′-tetramethylrhodamine (TAMRA) probe. The amplification assay included a negative control (water), a negative bacterial genomic control (DNA of *Leptospira* biflexa sv Patoc), and a positive control (DNA of *L. interrogans* sv Icterohaemorrhagiae), and the sample was tested in duplicate. The assay was performed on a 7900 High Throughput (HT) Fast Real-Time PCR System (Thermo Fisher Scientific) with the following thermal conditions: a hot-start step at 50 °C for 2 min, a holding step at 95 °C for 10 min and 45 cycles of 95 °C for 15 s and 60 °C for 60 s. Samples with cycle threshold (Ct) < 38 were considered positive. Samples having Ct values within the 38–40 range were considered doubtful, whereas samples having no FAM fluorescence signal or with Ct ≥ 40 were considered negative.

The genotyping characterization was performed on extracted DNA as previously described [11].

## 3. Results

### 3.1. Gross Pathology

**Figure 1:**
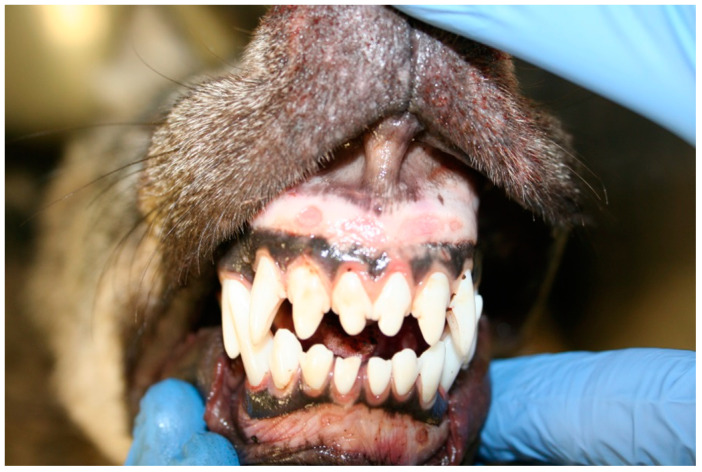
Erosive lesions in the upper and lower gingival.

**Figure 2:**
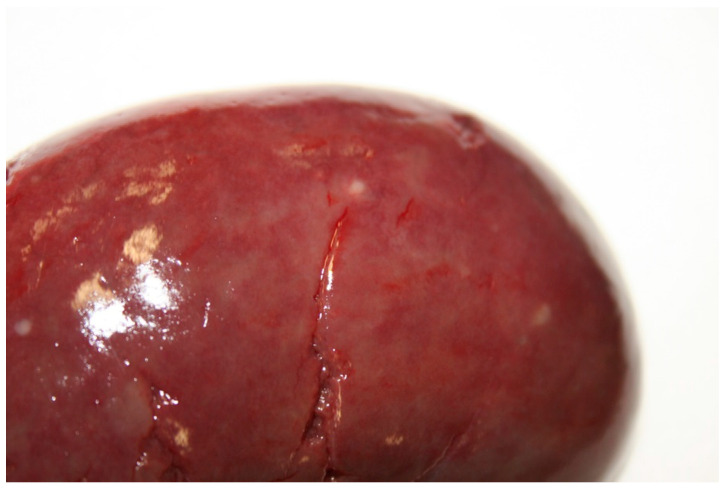
Diffusely congested kidney with white spotting cortical lesions.

Postmortem condition was adequate and nutritional condition was good. The wolf showed alopecic areas referable to seasonal changes; ectoparasites were not detected. Traumatic lesions included skin ecchymosis 0.5–1 cm diameter in the forelimbs and legs and subcutaneous hemorrhages 8 × 10 cm wide in the thorax and along the back, costal fractures, bleedings in the abdomen and thorax, representing the final cause of death. Erosive lesions of gingival mucosa, 0.5 cm diameter, in both upper and lower gingiva but more severe in the upper one were observed (Figure 1); we noticed mild liver enlargement and multifocal degenerative-necrotic areas along with hyperemic reactive lesions; the spleen was moderately enlarged and severely congested with focal hyperemic lesions; mesenteric lymph-nodes were moderately enlarged; kidneys appeared diffusely congested with white spotting cortical lesions (Figure 2); severe multifocal lung hemorrhages, mild subepicardial petechial hemorrhages and mild endocardial multifocal hemorrhages in the right auricle were present. The urinary bladder was empty. Grape skins, plastic elements, and mammal hairs were detected in the gastric lumen; catarrhal gastritis characterized by localized reddish mucosa in the fundic area and plicae was observed.

### 3.2. Diagnostic Tests

All virological investigations, including electron microscopy and specific tests for canine distemper virus, adenovirus, and parvovirus resulted negative. *E. multilocularis* and Trichinella were not detected. *Staphylococcus intermedius* was isolated from the liver and kidney, whereas *Salmonella* spp. was not detected. Although the liver presented advanced autolysis, histopathological kidney changes included severe multifocal interstitial nephritis and moderate multifocal vascular and interstitial congestion. The real-time PCR to detect pathogenic species of *Leptospira* performed on kidney samples was positive. Serological reactions for serogroup Gryppotyphosa serovar Gryppotyphosa (1:6400), Pomona serovar Pomona (1:800), and Icterohaemorrhagiae serovar Icterohaemorrhagiae (1:200) were evidenced by MAT. Molecular typing by MLST carried out on detected *Leptospira* characterized it as ST117. The inference with the Bacterial Isolate Genome Sequence Database (BIGSdb) [12] was used to define the species, the serogroup, and the serovar status, which resulted to be L. kirschneri Pomona Mozdok, respectively. The kidney sample was considered not suitable for the microbiological isolation in culture, as the carcass was in good condition, but not fresh enough to have the chance to find viable *Leptospira*. Mammal hairs detected in the gastric lumen were attributed to a probably scavenged domestic cat (*Felis silvestris catus*).

## 4. Discussion

The molecular typing performed directly on tissue samples allowed the identification of *Leptospira* genospecies, serogroup, and serovar, even in the absence of a strain isolated in pure culture. The microbiological isolation was not attempted as the subject was found dead in the evening and submitted to the gross pathology around 16–18 h later, so the tissue autolysis was started and the chances to find viable *Leptospira* were very slight. Regarding the serological response, in the case here described, the specific response to serogroup Pomona was present (1:800), even if the higher titer (1:6400) was towards the serogroup Gryppotyphosa. It is known that MAT, during the acute phase of infection, can give multiple cross-reactions. It was commonly considered that the serogroup responsible for infection is the one giving the higher antibody titer response, but the new tools nowadays available for diagnosis are sometimes showing different and surprising results. In another study performed in the same region [11], no samples positive to serogroup Grippotyphosa were found, despite a widespread serological response towards this serovar in dogs suffering from acute leptospirosis. Finally, we had the chance to highlight the serological response toward the antigen belonging to the serogroup Pomona, serovar Pomona and not to the serovar Mozdok, the real infectious strain. Even though it is well known that the immune response is serogroup-specific, probably the serovar-specific titer would have been higher.

Serovar Mozdok was previously detected in domestic pigs (*Sus scrofa*), cattle, and dogs [4,11,13,14] while *L. interrogans*, serogroup Pomona, serovar Pomona was evidenced by MAT in wild boars (*Sus scrofa*) [15]. In the same study, *L. kirschneri* was also reported in a wild boar [13]. Relationships at the interface of domestic and wild species including small rodents could be linked to prey-predator epidemiology and to direct or indirect contact. Although small mammals are generally not considered to have an important role in the epidemiology of the Pomona serogroup, in the eastern Croatia *L. kirschneri*, serogroup Pomona, serovar Mozdok was the most frequently isolated *Leptospira* in small rodents [16], and the sequence type ST117 was also detected in German small mammals [17]. In the close Slovenian wild boar population, serological assessment of seroprevalence was 45% for at least one serovar and 7% against Pomona serogroup [18]. In consideration of lacking data on *Leptospira* in wild boars in the study area, serological investigations in this species may give further insights into the epidemiology of leptospirosis. In Spain, the Iberian wolf (*Canis lupus signatus*) has been suggested as an environmental sentinel for serovars for which rodents, ungulates, and dogs are natural hosts [19]. In contrast, a survey including 43 wolf sera resulted negative in Central Italy [20]. High antibody titers evidenced in this individual may be suggestive of an acute and active phase of the infection. Another wolf almost 2 years old, probably belonging to the same pack, road-killed in February 2019 did not show pathological leptospiral induced changes and was PCR negative, but it was seropositive for serogroup Grippotyphosa serovar Grippotyphosa (1:200). Although *L. interrogans* has been more frequently associated with zoonotic transmission of leptospirosis, during the last decades, the role of *L. kirschneri* has been increasingly related to human and canine leptospirosis [21]. *L. kirschneri* appears to be the most common pathogenic species in Europe after *L. interrogans* [22].

It must be highlighted that serogroup Pomona is not currently included in commercial vaccines available in Italy; furthermore, Pomona infection in dogs has also been suggested of having a worse outcome compared to other serogroups [23]. Serovar Mozdok ST117 has been previously identified from dogs of the neighboring Veneto region, sometimes leading to a lethal outcome [4]. Gross pathological changes of this wolf were in accordance with similar findings in affected dogs [21], however, we did not find any description of pathological modifications due to *Leptospira* infection in the wolf in literature, although natural infection was reported [1]. Renal lesions are not frequently reported in European and Italian wildlife: in absence of macroscopic lesions, interstitial nephritis was histologically observed in foxes and mongooses (*Herpestes ichneumon*) in Spain [24] and chronic nephritis was described in red deer (*Cervus elaphus*) in central Italian Alps [25].

The gastric content of this wolf was clear evidence of the close interface with humans, human activities, and domestic animals. Therefore, the possible spill-over/spill-back of pathogens in general and on the occasion of emerging epidemics such as rabies and canine distemper virus should be taken into account.

Although this subject was not seen alive before the car killing, we cannot exclude that leptospiral infection may have induced clinical signs or behavioral changes, such as fever, weakness, or longer reaction times, prompting the accident.

Ongoing surveillance of wild carnivores gave and may provide further insights on leptospiral epidemiology.

## 5. Conclusions

Regardless of the role of *Leptospira* infection as an eventual predisposing factor to the road killing of this wolf, to the best of the authors’ knowledge, this is the first report of *Leptospira* induced pathology in a wolf in Europe. Although road accidents and poaching are generally recorded as the main mortality factors in this species, it is unclear if leptospirosis may represent a limiting factor in wolf demographic expansion in the area. There is no evidence that wolf populations may represent a reservoir of infection, nevertheless, leptospirosis in wolf arises issues at wildlife/domestic animals/human interface as well as conservational matters; therefore, surveys on *Leptospira* infection in free-ranging wildlife species should be pursued in order to achieve further epidemiological knowledge on *Leptospira* strains distribution.

## Data Availability

Data is contained within the article.
